# Supplementary material for: Maternal Choline Supplementation Modulates Placental Markers of Inflammation, Angiogenesis, and Apoptosis in a Mouse Model of Placental Insufficiency
Source: Nutrients. 2019 Feb 12;11(2):374. doi: 10.3390/nu11020374 (PMC6412879; doi:10.3390/nu11020374)
Supplement: Supplementary file 1 [file nutrients-11-00374-s001.zip › Supplemental Table 2.docx]

**Supplementary Material**

**Supplemental Table 2.** Embryo weight, placental weight, crown rump length, and placental efficiency and litter size in *Dlx3+/-* dams in response to three different maternal choline treatments (control, 2X and 4X) at E10.5, E12.5, E15.5 and E18.5. Embryo weight, placental weight, crown rump length, and placental efficiency were analyzed using mixed linear models controlling for maternal ID, fetal genotype, fetal sex, and litter size. Litter size analyzed using ANOVA.
**P* < 0.05 vs. 1X controls. #*P* < 0.1 vs. 1X controls. n=7-10 dams per treatment, per time point. Values are mean ± SEM.

| **Time point** | **Diet** | **Embryo Weight** | **Placental weight** | **Crown Rump Length** | **Placental Efficiency** | **Litter size** |
| --- | --- | --- | --- | --- | --- | --- |
| **E10.5** | **1X** | 0.009 ± 0.001 | 0.033 ± 0.002 | 4.42 ± 0.1 | 0.27 ± 0.03 | 7.2 ± 1.1 |
|  | **2X** | 0.011 ± 0.001 | 0.036 ± 0.002 | 4.46 ± 0.2 | 0.31 ± 0.03 | 8.3 ± 1.3 |
|  | **4X** | 0.015 ± 0.001* | 0.039 ± 0.002* | 4.92 ± 0.2* | 0.41 ± 0.04* | 7.0 ± 1.1 |
|  |  |  |  |  |  |  |
| **E12.5** | **1X** | 0.061 ± 0.004 | 0.053 ± 0.003 | 8.19 ± 0.2 | 1.20 ± 0.1 | 5.1 ± 1.0 |
|  | **2X** | 0.066 ± 0.004 | 0.051 ± 0.003 | 8.08 ± 0.2 | 1.28 ± 0.1 | 5.3 ± 1.1 |
|  | **4X** | 0.055 ± 0.004 | 0.049 ± 0.003 | 8.10 ± 0.2 | 1.14 ± 0.1 | 6.3 ± 1.0 |
|  |  |  |  |  |  |  |
| **E15.5** | **1X** | 0.381 ± 0.02 | 0.095 ± 0.004 | 14.73 ± 0.2 | 4.12 ± 0.3 | 4.9 ± 0.6 |
|  | **2X** | 0.417 ± 0.03 | 0.084 ± 0.005# | 14.87 ± 0.3 | 4.94 ± 0.3# | 3.8 ± 0.6 |
|  | **4X** | 0.386 ± 0.02 | 0.092 ± 0.004 | 14.91 ± 0.2 | 4.38 ± 0.3 | 5.8 ± 0.6 |
|  |  |  |  |  |  |  |
| **E18.5** | **1X** | 1.283 ± 0.03 | 0.101 ± 0.004 | 24.04 ± 0.6 | 13.03 ± 0.6 | 5.9 ± 0.9 |
|  | **2X** | 1.218 ± 0.03# | 0.096 ± 0.004 | 24.51 ± 0.6 | 12.93 ± 0.6 | 4.8 ± 0.8 |
|  | **4X** | 1.159 ± 0.03* | 0.098 ± 0.004 | 23.65 ± 0.6 | 12.29 ± 0.6 | 5.6 ± 0.9 |
