# Supplementary material for: Maternal Choline Supplementation Modulates Placental Markers of Inflammation, Angiogenesis, and Apoptosis in a Mouse Model of Placental Insufficiency
Source: Nutrients. 2019 Feb 12;11(2):374. doi: 10.3390/nu11020374 (PMC6412879; doi:10.3390/nu11020374)
Supplement: Supplementary file 1 [file nutrients-11-00374-s001.zip › Supplemental Table 1.docx]

**Supplementary Material**

**Supplemental Table 1.** Primers for genotyping and RT-qPCR.

| ***Gene*** | ***Name*** | ***Reference Sequence*** | ***Primer Sequences*** | ***Annealing Temperature*** |
| --- | --- | --- | --- | --- |
| ***Dlx3*** | Distal-less homeobox 3 | NC_000077.6 | F: 5’ GTGAACGGCAAGCCCAAA 3’  R: (Wild type allele)  5’ CTCTGTGACACGCCATACACAGTT 3’  R: (Knockout allele) 5’ AAAGGCCCGGAGATGAGGAAGAG 3’ | Touchdown from 63° to 49° |
| ***Sry*** | Sex determining region Y | NC_000087.7 | F: 5’ TGGGACTGGTGACAATTGTC 3’  R: 5’ GAGTACAGGTGTGCAGCTCT 3’ | 60°C |
| ***Vegfa*** | Vascular endothelial growth factor A | NM_001025250.3 | F: 5' CACTGGACCCTGGCTTTACT 3’  R: 5' ACTTGATCACTTCATGGGACTTCT 3’ | 63°C |
| ***Pgf (Plgf)*** | Placental growth factor | NM_008827.3 | F: 5' TGTGCCGATAAAGACAGCCA 3'  R: 5' TCGTCTCCAGAATAGGTCTGC 3' | 63°C |
| ***Eng*** | Endoglin | NM_007932.2 | F: 5' ATCAGTTTCCCGTCAGGCTC 3'  R: 5' GTTCGATGGTGTTGGATGCC 3' | 63°C |
| ***MMP14*** | Matrix metalloproteinase 14 | NM_008608.4 | F: 5' GCCCTCTGTCCCAGATAAGC 3'  R: 5' TTGGTTATTCCTCACCCGCC 3’ | 63°C |
| ***NfκB*** | Nuclear factor of kappa light polypeptide gene enhancer in B cells 1 | NM_008689.2 | F: 5' AGCAACCAAAACAGAGGGGA 3'  R: 5' TTTGCAGGCCCCACATAGTT 3’ | 60°C |
| ***Il1b*** | Interleukin 1 beta | NM_008361.4 | F: 5' TGCCACCTTTTGACAGTGATG 3'  R: 5' GCTCTTGTTGATGTGCTGCT 3’ | 60°C |
| ***Tnfα*** | Tumor necrosis factor alpha | NM_013693.3 | F: 5’ AAGTTCCCAAATGGCCTCCC 3’  R: 5’ TGGTGGTTTGCTACGACGTG 3’ | 60°C |
